# Evidence synthesis summary formats for clinical guideline development group members: a mixed-methods systematic review protocol

**DOI:** 10.12688/hrbopenres.13325.2

**Published:** 2022-05-10

**Authors:** Melissa K. Sharp, Barrie Tyner, Dayang Anis Binti Awang Baki, Cormac Farrell, Declan Devane, Kamal R. Mahtani, Susan M. Smith, Michelle O'Neill, Máirín Ryan, Barbara Clyne

**Affiliations:** 1 Health Research Board Centre for Primary Care Research, Department of General Practice,, Royal College of Surgeons in Ireland, Dublin, Ireland; 2 Health Information and Quality Authority, Dublin, Ireland; 3 School of Medicine, Royal College of Surgeons in Ireland, Dublin, Ireland; 4 School of Nursing and Midwifery, NUI Galway, Galway, Ireland; 5 Evidence Synthesis Ireland and Cochrane Ireland, Galway, Ireland; 6 Nuffield Department of Primary Care Health Sciences, University of Oxford, Oxford, UK; 7 Department of Pharmacology & Therapeutics, Trinity College Dublin, Dublin, Ireland

**Keywords:** presentation of findings, evidence summaries, summary of findings table, communication, mixed-methods systematic review

## Abstract

**Introduction**: Evidence syntheses, often in the form of systematic reviews, are essential for clinical guideline development and informing changes to health policies. However, clinical guideline development groups (CGDG) are multidisciplinary, and participants such as policymakers, healthcare professionals and patient representatives can face obstacles when trying to understand and use evidence synthesis findings. Summary formats to communicate the results of evidence syntheses have become increasingly common, but it is currently unclear which format is most effective for different stakeholders. This mixed-methods systematic review (MMSR) evaluates the effectiveness and acceptability of different evidence synthesis summary formats for CGDG members.

**Methods**: This protocol follows guidance from the Joanna Briggs Institute on MMSRs and is reported according to the Preferred Reporting Items for Systematic Reviews (PRISMA)-P guideline. A comprehensive search of six databases will be performed with no language restrictions. Primary outcomes are those relating to the effectiveness and preferences for and attitudes towards the different summary formats. We will include qualitative research and randomised controlled trials. Two reviewers will perform title, abstract, and full-text screening. Independent double-extraction of study characteristics and critical appraisal items will be undertaken using a standardised form. We will use a convergent segregated approach to analyse quantitative and qualitative data separately; results will then be integrated.

**Discussion**: The results of this systematic review will provide an overview of the effectiveness and acceptability of different summary formats for evidence synthesis findings. These findings can be helpful for those in or communicating to guideline development groups. The results can also inform the development and pilot-testing of summary formats for evidence summaries.

## Introduction

Clinical guidelines support decision making to improve patient outcomes and quality of care in a cost-effective manner ^1^. The development of a clinical guideline involves a rigorous synthesis of the best available evidence on a specific clinical topic. It may involve formal consensus methods with a range of multidisciplinary stakeholders ^2– 5^. Guideline development groups comprise a range of decision makers, often including healthcare professionals, methodologists, health policymakers, clinicians, and patient representatives – all of whom have varying levels of expertise in evidence synthesis methods. This complicates the consensus process as stakeholders may prioritise and understand the findings of evidence syntheses, such as systematic reviews, differently ^6^.

While the methods and recognition of the importance of systematic reviews have advanced in recent decades ^7, 8^, there are still barriers to their creation and use ^9, 10^. A meta-analysis of nearly 200 systematic reviews registered on the international Prospective Register of Systematic Reviews (PROSPERO) registry found that the average systematic review, from registration to publication date, takes 67.3 weeks, involves an average of five authors, and requires the full-text screening of 63 papers (range: 0–4385) ^9^. The number of academic papers and systematic reviews being published in recent decades has rapidly increased ^11– 13^, further accelerating during the recent COVID-19 (coronavirus disease) pandemic ^14^. The expanding evidence base and acceptance of trade-offs in validity in time-sensitive matters ^15^, has resulted in the growing popularity of other evidence synthesis methods, such as rapid reviews ^16^. This increase in different types of evidence synthesis methods further complicates matters for guideline development groups, who may interpret different types of systematic reviews in different ways based on how familiar they might be with particular approaches.

For those using different types of evidence synthesis to inform clinical guideline development and health policy, the amount of included studies, length, and technical nature of evidence syntheses can make it difficult to find answers about the effectiveness of healthcare interventions ^10, 17^. Previous work has highlighted that decision makers more easily understand evidence summaries than complete systematic reviews ^18, 19^. These summaries can come in a variety of different formats such as policy briefs, one-page reports, abstracts, summary of findings tables, plain language summaries, visual abstracts or infographics, podcasts, and more. While formatting may vary, decision-makers have expressed several key preferences, such as succinct summaries highlighting contextual factors like local applicability and costs ^17, 20^.

Succinctness should be inherent in an evidence summary, but how this distilled information is formatted and presented affects the interpretation and use of systematic reviews ^21^. It is currently unclear which evidence summary format is most helpful for decision making for different guideline development group stakeholders. For example, Cochrane recommends a ‘summary of findings’ table ^7^ but testing with users familiar with the Cochrane library and evidence-based practices raised concerns around comprehension and presentation of results and the balance between precision and simplicity ^22^. Others have tested the presentation of information using different formats such as an abstract, plain-language summary, podcast or podcast transcription with no clear answer regarding which format was most suited to which stakeholder and resulted in the best understanding ^23^. Similarly, infographics, plain-language summaries, and traditional abstracts were found to be equally effective in transmitting knowledge to healthcare providers; however, there were differences in measures of acceptability (i.e., user-friendliness and reading experience) ^24^.

To better support clinical guideline development groups and decision-makers, it is important to identify which format works best for which stakeholder. Previous reviews have focused on identifying barriers and facilitators to use, or have been solely based on summary of findings tables ^10, 25^. As impacts on decision-making and preferences for formats may be evaluated through different study designs, a comprehensive synthesis of the evidence is needed beyond a typical single method systematic review. Mixed methods systematic reviews (MMSR) can more easily identify discrepancies within available evidence, pinpoint how quantitative or qualitative research has focused on particular interest areas, and offer a deeper understanding of findings ^26^. A MMSR is especially useful for this project as it brings together findings of effectiveness and experience so findings are more useful for decision makers ^27^. Guideline developers need to consider diverse considerations in their work such as feasibility, priority, cost effectiveness, equity, acceptability, and patient values and preferences ^28, 29^. Similarly, a MMSR allows us to consider and integrate data from a variety of different questions and synthesize information in a single project.

### Objectives

The aim of this mixed methods systematic review is to evaluate the effectiveness of, preferences for, and attitudes towards, different communication formats of evidence summary findings amongst guideline development group members, including healthcare providers, policy makers and patient representatives. To achieve this, the proposed MMSR will answer the following questions:

1. How and to what degree do different summary formats (digital, visual, audio) of presenting evidence synthesis findings impact the end user’s understanding of the review findings?
2. What are the end users’ preferences for and attitudes towards these formats?

## Protocol

The proposed systematic review will be conducted in accordance with the Joanna Briggs Institute (JBI) Manual for Evidence Synthesis which details the methodology for mixed methods systematic reviews (MMSR) ^26^.

### Eligibility criteria

 As this is a MMSR, we will include quantitative (i.e., randomised controlled trials), qualitative, and mixed methods studies evaluating the effectiveness and/or preferences for and attitudes towards evidence summary formats. We will exclude conference abstracts, case reports, case series, editorials, and letters. Further details regarding eligibility criteria are given within the review-relevant sections below.

 We are interested in studies involving stakeholders such as policy makers, healthcare providers, and health systems managers, as well as other GDG members such as clinicians, patient representatives, and methodologists such as systematic review authors. We will exclude studies where the sole participants are students, the general population (those not involved in the clinical guideline development process), and journalists as communication to these populations is more complex given a wide variety of confounding factors. We will also exclude studies related to clinical decision-making for individual patients.

We have followed the Population, Intervention, Comparison, Outcome (PICO) format for the quantitative review ( Table 1) and the Sample, Phenomenon of Interest, Design, Evaluation, Research type (SPiDER) format for the qualitative review ( Table 2) and will present unique aspects of each methodological approach within the relevant sections below.

**Table 1.** PICO for the quantitative review of effectiveness.

**P**opulation	Members of guideline development groups (e.g., policy makers, decision makers, healthcare professionals, methodologists, patient representatives)
**I**ntervention	A summary format which communicates evidence synthesis findings
**C**omparator	Alternative summary formats
**O**utcomes	Quantitative estimates of effectiveness and acceptability

**Table 2.** SPiDER for the qualitative evidence synthesis.

**S**ample	Members of guideline development groups (e.g., policy makers, decision makers, clinicians, methodologists, patient representatives)
**P**henomenon of **i**nterest	How summary formats impact decision-making and understanding of evidence synthesis findings
**D**esign	Focus groups, interviews, questionnaires, open-ended survey responses
**E**valuation outcomes	Views, attitudes, opinions, experiences, perceptions, beliefs, feelings, understanding
**R**esearch type	Qualitative studies and mixed-methods studies with primary qualitative data collection

***Quantitative systematic review*.** Due to the complexity of stakeholders, evidence synthesis types, and summary formats, there is a high potential that confounding factors will be extensive. Relatedly, randomised controlled trials (RCTs) are the most appropriate design to evaluate the effectiveness of the interventions in question. Thus, we chose to restrict to RCTs (e.g., parallel, crossover, cluster, stepped-wedge, etc.) only in order to focus on the performance and impact of summary formats in optimal settings. We will include studies where the intervention is any summary mode (e.g., visual, audio, text-based, etc.) which communicates the findings from an evidence synthesis study (e.g., systematic review, qualitative evidence synthesis, rapid review, etc.) to policy-makers and decision makers, including guideline development groups (GDGs). We anticipate that included summary formats may encompass visual abstracts, Summary of Findings tables, one-page summaries, podcasts, Graphical Overview of Evidence Reviews (GofER) diagrams, and others. We will not exclude a summary format if it is one that we did not explicitly list in our search strategy ( Table 3). Studies in which the summaries are one component of a multi-component intervention will be excluded, as will decision aids for direct patient care.

**Table 3.** Ovid MEDLINE search strategy.

	Ovid MEDLINE(R) and Epub Ahead of Print, In-Process, In-Data-Review & Other Non-Indexed Citations, Daily and Versions(R) 1946 to April 13, 2021	Search results
1	exp Administrative Personnel/	41017
2	((health OR healthcare OR hospital*) ADJ2 (administrator* OR analyst* OR decisionmak* OR decision-mak* OR manager* OR official* OR policymak* OR policy-mak* OR policy OR policies OR provider)).tw.	73550
3	exp Decision Making/ OR Exp Policy Making/ OR exp Health Policy/	332820
4	((decision* OR policy OR policies) ADJ2 (analys* OR analyz* OR maker* OR making OR develop*)).tw.	213763
5	(analyst* OR clinician OR decision-mak* OR decisionmak* OR doctor OR guideline development group* OR advisory group OR knowledge user* OR knowledge-user* OR policy-mak* OR policymak* OR stakeholder* OR stake-holder* OR stake holder* OR end user* OR end-user*).tw.	359621
6	1 OR 2 OR 3 OR 4 OR 5	731506
7	exp Evidence-Based Practice/ OR exp "Review Literature as Topic"/ OR meta-analysis as topic/ OR exp Technology Assessment, Biomedical/	128395
8	(knowledge ADJ2 synthes*).tw.	1051
9	(meta*) ADJ2 (analysis OR regression OR review OR overview OR synthes*)	251572
10	meta-analy* OR meta-regression OR meta-review* OR meta-synthes* OR megasynthes*	231916
11	(evidence) ADJ2 (synthes* OR summar*)	20882
12	(quantitative OR qualitative OR systematic OR rapid OR scoping OR realist OR Cochrane OR evidence) ADJ2 (review* OR overview*)	270363
13	HTA OR health technology assessment	6744
14	7 OR 8 OR 9 OR 10 OR 11 OR 12 OR 13	530362
15	exp Data Visualization/ OR exp Health communication/ OR exp Implementation science/	3579
16	summary of findings OR summary-of-findings OR table* OR tabular	156130
17	plain-language summar* OR plain language summar*	1758
18	infographic* OR podcast* OR visual abstract* OR fact box* OR summary format OR blogshot OR blog shot OR podcast OR video OR GRADE evidence profile OR policy brief OR league table* OR bulletin OR infogram or 1-page summary OR SUPPORT summary OR brief* or summar* OR graphic* OR audio	1011424
19	(communicat* OR presentat*) ADJ2 (finding*)	2500
20	15 OR 16 OR 17 OR 18 OR 19	1156243
21	perceive OR understand OR understanding OR acceptability OR effectiveness OR efficacy OR satisfaction OR usability	2703824
22	usefulness OR credibility OR clarity OR comprehensive OR appeal OR appropriateness OR preference$	693195
23	21 OR 22	3247866
24	6 AND 14 AND 20 AND 23	3830

For studies examining the effectiveness of evidence summary formats, we will include any comparison to an alternative active comparator. Studies where the comparison is no intervention (e.g., the plain full-text of a manuscript) will be excluded. We do not anticipate finding evidence syntheses with no form of summary or abstract as international organisations, journals, and reporting guidelines would consider a summary to be a mandatory component of any report or peer review manuscript produced.

Our primary outcomes of interest are:

1. Effectiveness
  a. User understanding and knowledge, and/or beliefs in key findings of evidence synthesis (e.g., changes in knowledge scores about the topic included in the summary)
  b. Self-reported impact on decision‐making
  c. Intervention metrics (e.g., the time needed to read the summary, expressed language accessibility issues or scale scores)

2. Acceptability
  a. Preferences and attitudes (e.g. Likert scales reporting user satisfaction, perceptions, readability).

We will not be including outcomes related to health literacy, numeracy, nor risk communication in patient-centred care. We are aligning our definition of ‘health literacy’ with a recent systematic review on its meaning, which is complex in nature and composed of ‘(1) knowledge of health, healthcare and health systems; (2) processing and using information in various formats in relation to health and healthcare; and (3) ability to maintain health through self-management and working in partnerships with health providers.’ As impacts on one’s individual health or clinical care is not the main focus of this review, we are focusing only on one aspect (2) -- impacts on one’s understanding of knowledge which is constrained to a specific topic that an evidence summary is covering ^30^.

***Qualitative evidence synthesis*.** Primary studies investigating the understanding and acceptability of evidence summary formats will include qualitative studies (e.g, interviews or focus groups). Mixed-methods studies with primary qualitative data collection will be included if they meet the inclusion criteria for a randomised controlled trial and where it is possible to extract the findings derived from the qualitative research. We prioritized the inclusion of qualitative data from primary studies over free text from questionnaire surveys as we hypothesized primary data would be richer and thicker and thus more informative.

 Our primary outcomes of interest relate to participant’s views and experiences with summary formats. This includes their perceptions of the impact of summary formats on their understanding, knowledge, and decision making, and participant’s beliefs, attitudes, and feelings towards usability and readability. 

### Information sources and search strategy

The following databases will be searched from inception to May 2021: Ovid MEDLINE, EMBASE, APA PsycINFO, CINAHL (Cumulative Index to Nursing and Allied Health Literature), Web of Science, and Cochrane Library. The search strategy for Ovid MEDLINE includes a combination of keywords and medical subject headings (MeSH) terms for GDG members, evidence syntheses, and formats for the communication of findings (see Table 3). As we are looking for primary research on the impacts or effects of interventions and attitudes towards them, we do not anticipate that this literature will be found in grey literature sources such as government or agency websites. Additionally, it is anticipated that controlled trials will have short time points of assessment (and follow-up) thus we do not believe that searching registries will benefit our study. This search strategy has been informed by the strategies of similar reviews in the same topic area ^10, 25^. Aligned with the Peer Review of Electronic Search Straggles (PRESS) Statement ^31^, we engaged a medical librarian after the MEDLINE search was drafted but before it was translated to the other databases. As we are including a range of study designs, we did not apply study design specific filters. Although we have used a PICO and SPiDER approach for the quantitative and qualitative reviews we used the PICO format to inform the search strategy as previous researchers found that the SPiDER approach for search strategies may be too restrictive and specific ^32^. Language and date restrictions will not be applied.

Backwards citation identification on all eligible studies will be performed using the citationchaser Shiny application built in R version 1.4 ^33^. This application performs backwards citation screening (reviewing reference lists) and internally de-duplicates results. Each step of the search is summarised for transparency and references are given as a downloadable RIS file.

### Data management and selection process

All citations will be downloaded and stored in Zotero reference manager version 5.0. For ease, rather than using Zotero for screening, title and abstract screening will be managed using Covidence. Two reviewers will independently screen titles and abstracts for inclusion criteria. Disagreements for inclusion will be resolved through discussions. If it is still unclear if the paper should be included, both authors will review the full version of the paper and discuss it again. If there is still disagreement, a third review author will be consulted. The screening process will be documented in the final manuscript using the Preferred Reporting Items for Systematic Reviews (PRISMA) flow diagram ^34^ and a supplemental file detailing the reason for exclusion for each individual study will be made publicly available.

### Data collection

Two review authors will independently extract data from each of the included studies using a standardised data-extraction form. If there are disagreements or discrepancies, the two authors will discuss and consult with a third review author if needed. Where possible, qualitative outcomes such as themes and categories will be extracted into the standardized form. In parallel, articles containing qualitative methods will be also imported in NVivo12 for line-by-line coding for information related to outcomes. This separate but parallel data extraction is important for our analytical approach of the qualitative data which is discussed in greater detail in the Qualitative Analysis section. The following information will be extracted using the pilot-tested standardized data-extraction form

- Bibliometric data (first author, title, journal, year of publication, language)
- Study characteristics (setting, participants demographics, country, study design, intervention, comparators, theoretical framework, analytical approach)
- Intervention characteristics will be collected following the structure of the Template for Intervention Description and Replication (TiDieR) checklist ^35^ to provide detailed information on the why, what, who, how, where, and when of the intervention described.
- Primary and secondary outcomes (quantitative estimates of effectiveness and acceptability; qualitative expressions of views, attitudes, opinions, experiences, perceptions, beliefs, feelings, and understanding)
- Data from the domains listed within the JBI critical appraisal tools for qualitative and quantitative studies
- Funding sources

If information is missing from the study report, we will contact authors to inquire about these gaps. We will provide narrative syntheses in lieu of imputing missing data.

### Bias and quality assessments

The JBI critical appraisal checklists will be used to assess the individual randomized controlled trials and qualitative studies. Two review authors will independently complete the critical appraisal checklist for each included study. Differences will be resolved through discussion and consultation with a third review author if necessary. These checklists will provide useful contextual information about the included studies such as information about performance bias. Checklist items cover things like intervention assessors and their reflexivity which are important factors to consider as participant attitudes towards summary formats may be influenced by external factors such as who created the summary (e.g., their own vs. an external organisation).

An assessment of the overall certainty of evidence using the GRADE or ConQual approach is not recommended for a JBI MMSR26. This is due to the complexities in the analysis wherein the data from separate quantitative and qualitative evidence is transformed and integrated.

If quantitative data allows for a meta-analysis, a forest plot will be generated using R. If we find a low number of studies, large treatment effects, few events per trial, or all trials are of similar sizes, we will use the Harboard test for publication bias ^36^ as it reduces the false positive rate. Egger’s test ^37^ for funnel plot asymmetry will be used to investigate small study effects and publication bias.

### Quantitative analysis

A narrative synthesis will be performed, however, if appropriate, quantitative data from randomised control trials will be synthesised using meta-analysis. Heterogeneity will first be explored by assessing pertinent study characteristics that may vary across the included studies (i.e., participant group, or summary format type). If sufficient data is available, subgroup analyses (e.g., participant groups such as medical professionals versus policy makers or intervention type such as visual abstracts versus plain abstracts) will be conducted. Furthermore, statistical heterogeneity will be explored according to statistical guidance on heterogeneity ^7^, an estimated I ^2 ^of 50–90% represents substantial heterogeneity. We will weigh this against an χ ^2^ test for heterogeneity (<.10). If our results indicate 50% or greater and a low χ ^2^ statistic, this indicates that the heterogeneity may not be due to chance, thus we will not pool results into a meta-analysis. If data can be pooled, effect sizes and accompanying 95% confidence intervals will be reported as either relative risks (for dichotomous and dichotomised ordinal data) or standardized mean differences (for continuous data). Where data is available, we will compare and contrast reported findings on preference and whether or not preference is aligned with improvement of outcomes of impact such as knowledge.

### Qualitative analysis

Where possible, qualitative findings will be pooled together using the meta-aggregation approach, which allows a reviewer to present findings of included studies as originally intended by the original authors ^38^. This approach organises and categorises findings based on similarity in meaning and avoids re-interpretation. Therefore, it does not violate paradigms and approaches used by the original study authors. This approach also enables meaningful generalizable recommendations for practitioners and policy makers ^39^. If we are unable to pool findings together (i.e., create and present categories), likely due to an insufficient number of studies identified, a narrative summary will be presented.

### Mixed methods synthesis

Following JBI guidance for MMSR, we will use a convergent segregated approach that conducts separate quantitative and qualitative syntheses separately but at the same time and then integrates the findings of each ^26, 40^. The segregated design integrates evidence through a method called configuration which essentially arranges complementary evidence into a single line of reasoning ^26^. After the separate quantitative and qualitative analyses are conducted, they will be organized into a coherent whole, as they cannot be directly combined nor can one refute the other ^26, 41^. Converging or complementary data assumes that, while the streams of evidence may ask different research questions, they are related to different aspects or dimensions of the same phenomenon of interest ^26^. Data will be triangulated during the interpretation stage, comparing quantitative and qualitative findings side-by-side to identify areas where there is convergence, inconsistency, or contradiction in the data. We do not aim to transform the qualitative data into quantitative, nor vice versa. 

There are several methods for integrating qualitative and quantitative evidence syntheses ^26, 42^ in a convergent segregated MMSR. We will use a thematic synthesis method for integration which groups together similar codes, develops descriptive categories (or themes) to create an overall summary of findings ^43, 44^. Initial coding will be performed independently by two authors who will meet and discuss similarities and differences in coding to start grouping them into descriptive categories. A drafted summary of findings will be created by one author, reviewed by both, and discussions will be held until a final version is agreed upon. Two authors will discuss the descriptive categories and, as a group, will draft the final analytical categories with accompanying detailed descriptions.

If we have a sufficient number of included studies for meta-analysis (minimum of three), we will report information according to participant subgroups (e.g., clinicians versus policy makers), and outcomes (e.g., understanding, acceptability, etc.).

### Registration and amendments

As the focus of this review is not evaluating health-related interventions nor outcomes, we will not register the protocol on PROSPERO. However, we will preregister the study on Open Science Framework. If an amendment to this protocol is necessary, the date of each amendment will be given alongside the rationale and description of the change(s). This information will be detailed in an appendix accompanying the final systematic review publication. Changes will not be incorporated into the protocol.

### Dissemination of information

Findings will be disseminated as peer-reviewed publications. Data generated from the work proposed within this protocol will be made available on the aforementioned OSF project page.

## Discussion

This review will summarise the evidence on the effectiveness and acceptability of different evidence synthesis summary formats. By including a variety of evidence summary types and stakeholder participants, results can help tease apart the real-world complexity of guideline development groups and provide an overview of what summary formats work for which stakeholders in what circumstances. It is expected that review findings can support decision-making by policy-makers and GDGs, by establishing the best summary formats for presenting evidence synthesis findings.

## Data availability

No data are associated with this article.

### Reporting guidelines

OSF: PRISMA-P checklist for ‘Evidence synthesis summary formats for clinical guideline development group members: a mixed-methods systematic review protocol’. https://doi.org/10.17605/OSF.IO/SK4NX ^45^

Data are available under the terms of the Creative Commons Attribution 4.0 International license (CC-BY 4.0).
