# Supplementary material for: Evidence synthesis summary formats for clinical guideline development group members: a mixed-methods systematic review protocol
Source: HRB Open Res. 2022 May 10;4:76. Originally published 2021 Jul 15. [Version 2] doi: 10.12688/hrbopenres.13325.2 (PMC9433911; doi:10.12688/hrbopenres.13325.2)
Supplement: Peer review history

# Peer review: Reviewer response for version 2

**DOI:** 10.21956/hrbopenres.14775.r32053

**Published:** 2022-08-31

**Authors:** Karin Hannes

**Affiliations:** 1 Research group SoMeTHin'K (Social, Methodological and Theoretical Innovation Kreative), CESO, Faculty of Social Sciences, KU Leuven, Leuven, Belgium

I am happy with the responses of the authors.

Is the study design appropriate for the research question?

Yes

Is the rationale for, and objectives of, the study clearly described?

Yes

Are sufficient details of the methods provided to allow replication by others?

No

Are the datasets clearly presented in a useable and accessible format?

Not applicable

Reviewer Expertise:

NA

I confirm that I have read this submission and believe that I have an appropriate level of expertise to confirm that it is of an acceptable scientific standard.

# Peer review: Reviewer response for version 1

**DOI:** 10.21956/hrbopenres.14507.r29873

**Published:** 2022-02-15

**Authors:** Ivan Buljan

**Affiliations:** 1 Department of Research in Biomedicine and Health, University of Split School of Medicine, Split, Croatia

Thank you for the opportunity to review this interesting protocol. 

 Overall, the research plan is sound and described in detail. I have only a few minor suggestions to propose to make the protocol more precise.

 In Methods sections, under Eligibility criteria, I propose that you list all research designs that will be included in the analysis. Currently, only RCT-s are included, but it is not stated will all types of RCT-s will be included (parallel, crossover, etc). On top of that, what about non-randomized trials and control before and after studies? Will those studies be included, too?

 The sentence: "We will not be including outcomes related to health literacy, numeracy, nor risk communication in patient-centred care." is a bit problematic. Based on my understanding, health literacy is the examination of knowledge and/or understanding of health information, which will be the case of most research studies you include in the review. Maybe you wanted to state that you will not include studies that assessed health literacy with specific tests of health literacy?

 Would you consider including Open Science Framework registry as one of the databases in which you would perform a literature search?

**Minor points:**

- "If we have a sufficient number of included studies, subgroup analyses will be performed to investigate differences based on participant groups (e.g., clinicians versus policy makers) and outcomes (e.g., understanding, acceptability, etc.)" What would be considered a sufficient number of studies?

I hope those comments will help you in the process.

Is the study design appropriate for the research question?

Yes

Is the rationale for, and objectives of, the study clearly described?

Yes

Are sufficient details of the methods provided to allow replication by others?

Partly

Are the datasets clearly presented in a useable and accessible format?

Not applicable

Reviewer Expertise:

Public health; Health communication

I confirm that I have read this submission and believe that I have an appropriate level of expertise to confirm that it is of an acceptable scientific standard.

# Response

**Published:** 2022-03-31

**Authors:** Melissa Sharp

**Affiliations:** Royal College of Surgeons in Ireland, Ireland

Thank you for the opportunity to review this interesting protocol.

 Overall, the research plan is sound and described in detail. I have only a few minor suggestions to propose to make the protocol more precise.

*Author’s response: Thank you for the time spent reviewing our manuscript and for your valuable feedback.*

 In Methods sections, under Eligibility criteria, I propose that you list all research designs that will be included in the analysis. Currently, only RCT-s are included, but it is not stated will all types of RCT-s will be included (parallel, crossover, etc). On top of that, what about non-randomized trials and control before and after studies? Will those studies be included, too?

*Author’s response: Thank you for this question. We have edited the text to explicitly given examples of the types of RCTs that will be included ‘(e.g., parallel, crossover, cluster, stepped-wedge, etc.)’  The other reviewer had a related question regarding inclusion of different study designs. We have chosen to restrict study designs for this review to randomized controlled trials and qualitative studies as we believe these study designs are best able to either 1) control for confounding/interacting factors that could influence one’s attitude towards a format, such as who is disseminating the message (RCTs) or 2) better able to fully explore multiple factors influencing one’s perception (qualitative studies). We chose not to include non-randomised designs or observational studies due to the complexity of factors that influence one’s attitude towards a format. These study designs also relate directly to the objectives of the review (i.e., effectiveness and preferences for/attitudes towards formats).*

 The sentence: "We will not be including outcomes related to health literacy, numeracy, nor risk communication in patient-centred care." is a bit problematic. Based on my understanding, health literacy is the examination of knowledge and/or understanding of health information, which will be the case of most research studies you include in the review. Maybe you wanted to state that you will not include studies that assessed health literacy with specific tests of health literacy?

*Author’s response: Thank you for this comment. We believe that health literacy outcomes are quite distinct from knowledge/understanding outcomes. We recognize that the definition of health literacy has evolved over time and can often mean different things to different people so we have added additional information to the end of the outcomes section to clarify the focus of our project and the outcomes we are including. We have added text to the manuscript to clarify ‘We are aligning our definition of ‘health literacy’ with a recent systematic review on its meaning, which is complex in nature and composed of ‘(1) knowledge of health, healthcare and health systems; (2) processing and using information in various formats in relation to health and healthcare; and (3) ability to maintain health through self-management and working in partnerships with health providers.’ As impacts on one’s individual health or clinical care is not the main focus of this review, we are focusing only on one aspect (2) -- impacts on one’s understanding of knowledge which is constrained to a specific topic that an evidence summary is covering. [1]*

 Would you consider including Open Science Framework registry as one of the databases in which you would perform a literature search?

*Author’s response: Thank you for this suggestion.* *We did not search Open Science Framework as this registry is more often suited for observational studies and qualitative study protocols are still rare in the published literature. The search functionality of OSF (using Lucene) is also quite limited. For example using ‘evidence synthesis summary formats’ would result in over 23,000 registrations to screen. We did not search any registries for this study and will add this as a limitation in the discussion section of the final manuscript detailing the MMSR findings.*

 Minor points:

 "If we have a sufficient number of included studies, subgroup analyses will be performed to investigate differences based on participant groups (e.g., clinicians versus policy makers) and outcomes (e.g., understanding, acceptability, etc.)" What would be considered a sufficient number of studies?

*Author’s response: Thank you for this question. To our knowledge there is no numeric threshold for subgroup analyses for a MMSR. We edited the text to say ‘If we have a sufficient number of included studies for meta-analysis (minimum of 3), we will report information according to participant subgroups (e.g., clinicians versus policy makers), and outcomes (e.g., understanding, acceptability, etc.).’ as the analysis will be descriptive anyway.*

# Peer review: Reviewer response for version 1

**DOI:** 10.21956/hrbopenres.14507.r29872

**Published:** 2021-09-20

**Authors:** Karin Hannes

**Affiliations:** 1 Research group SoMeTHin'K (Social, Methodological and Theoretical Innovation Kreative), CESO, Faculty of Social Sciences, KU Leuven, Leuven, Belgium

This is a review underpinned by clear and interesting questions, that warrant a mixed method strategy. There is room for improvement though on the level of motivating choices for concepts and finetuning methods. 

 The biggest issue I have is on the relation between content and form in the choice for the mediums to be compared. The authors tend to compare forms (?), but attitude towards form is not fully independent to the way messages are framed, nor by whom they are disseminated. How do the authors intend to deal with this in their comparison?  (e.g. people might prefer a form such as video but it might fail to bring the message across, as participants are distracted by the form). 

 I was not fully sure why things like surveys measuring attitudes and opinions of people were not taken along in this review. (I am aware of the fact that guidance on the inclusion of such designs in reviews is scarce though-consider it a free offer to work towards a more comprehensive review type). Particularly, because in the RCTs authors tend to rely on self-reported measures, with LIKERT scales. Here, a comparison could sit with time, rather than comparator. Does people's attitude change when exposed to different formats over time? 

 Sometimes the arguments are not fully clear. In the introduction, attention is paid to problems complicating the conduct of systematic reviews. This is not the core focus of the project. The project is linked to the translation phase of turning review findings into evidence summaries. This is a matter of content, form and channels rather than the SR pre-track issues. It would therefore be more appropriate to highlight the diversity of actors involved in producing guidelines in relation to how communication and dissemination channels have changed because of that. I am a big fan of the authors discussing things like podcasts and multimedia accounts as examples. Naturally, this is linked to a society moving into multimodality and the limitations of numbers and narratives in reaching out to or working with different publics. 

 The PICO would perhaps work better if clinicians is changed into professionals. It would make it relevant to a broader target group.  Also, the comparator could also be no summary at all, for example, reading findings from reviews straight into the guideline procedure. Why are these explicitly excluded from the review?

 Do the authors only include qualitative studies where an intervention is evaluated by participants in terms of meaningfulness or applicability? Or do they also include qualitative studies where attitudes, viewpoints and options for form/channel/content are discussed from a more theoretical point of view, or in pilot cases? It is not clear where the selection sits on the qualitative side, but the data collection part suggests the first option. 

 Perhaps the authors may want to bring a rationale for why they want to limit the formats to textual and audio-visual productions, as there are many more forms available through which evidence is and can be communicated (such as installations, theatre, etc.) Perhaps these forms haven't been studied extensively in the context of guidelines, but in a protocol phase we are not sure what to find yet :-). 

 I would find it more practical if the authors would discuss their position towards critical appraisal and how to structure this instead of putting it in the data collection part as an extraction category (it is not clear what will actually be extracted for what purpose). Also, critical appraisal checklist for primary studies have a different purpose than GRADE and CONQUAL that work on a review level (which is clearly not the focus here when talking about the pool of studies included). I think the authors need to spell the two out in more detail, as they are used in different phases of the review process. 

 Would quality of a study not rather fit a sensitivity analysis rather than a subgroup analysis? What purpose would it have as a subgroup in the review? It has disappeared in the mixed method part as a subgroup analysis, so wouldn't mention it as a subgroup in other parts of the text.

 What do the authors mean with the phrase "If textual pooling is not available, a narrative summary will be presented." Is a narrative summary not already a textual pooling? Unclear. 

 While configuration might have different meanings, it is generally not commonly used as a metaphor for a separate type of synthesis. Configuration would make the different parts unrecognizable in the whole. I don't think meta-aggregation would achieve this level of integration. Perhaps integration is better than configuration here. 

 Also, thematic synthesis is not the same as meta-aggregation, and line by line coding is certainly not a part of meta-aggregation. I believe the authors need to make an informed choice between qualitative evidence synthesis approaches and then follow the guidance through. 

Is the study design appropriate for the research question?

Yes

Is the rationale for, and objectives of, the study clearly described?

Yes

Are sufficient details of the methods provided to allow replication by others?

No

Are the datasets clearly presented in a useable and accessible format?

Not applicable

Reviewer Expertise:

Methods

I confirm that I have read this submission and believe that I have an appropriate level of expertise to confirm that it is of an acceptable scientific standard, however I have significant reservations, as outlined above.

# Response

**Published:** 2022-03-31

**Authors:** Melissa Sharp

**Affiliations:** Royal College of Surgeons in Ireland, Ireland

This is a review underpinned by clear and interesting questions, that warrant a mixed method strategy. There is room for improvement though on the level of motivating choices for concepts and finetuning methods.

*Author’s response: Thank you for your review and thoughtful suggestions for our manuscript.*

 The biggest issue I have is on the relation between content and form in the choice for the mediums to be compared. The authors tend to compare forms (?), but attitude towards form is not fully independent to the way messages are framed, nor by whom they are disseminated. How do the authors intend to deal with this in their comparison?  (e.g. people might prefer a form such as video but it might fail to ring the message across, as participants are distracted by the form).

*Author’s response: Thank you for this comment. This is an interesting point, the studies we include in our review may well demonstrate a disconnect between what formats participants prefer, and what formats may actually produce improvement in outcomes such as knowledge. If included studies present this data we will be able to explore this more detail in our analysis but we suspect from the studies we have seen that this level of detail will not be available. Based on the importance of this suggestion, we will include this as a point of consideration in the discussion in the main manuscript in the protocol, we have made the following amendments to clarify our position on this:*

- *We have added additional information to the ‘bias and quality assessments’ section in the methods regarding this point: ‘These checklists will provide useful contextual information about the included studies such as information about performance bias. Checklists items cover things like intervention assessors and their reflexivity which are important factors to consider as participant attitudes towards summary formats may be influenced by external factors such as who created the summary (e.g., their own vs. an external organisation).’ *
- *We agree that the issue is complex and there are many confounders and interacting factors that could influence one’s attitude towards a format. Relatedly, we have chosen to restrict study designs for this review to randomized controlled trials and qualitative studies as we believe these study designs are best able to control for factors such as who is disseminating the message (RCTs) and are better able to fully explore multiple factors influencing one’s perception (qualitative studies). For example, these attitudes can be more fully explored with probes during semi-structured interviews. We have included the use of assessment tools for bias to further investigate issues such as the potential influence of the study assessors (e.g., those who designed or created the format) and their recipients (i.e, the readers/participants). We have also kept our outcomes broad so as to not take a one-dimensional view towards preference for one format over another. *
- *We can describe differences between preferences for formats and quantitative outcomes such as knowledge in the analysis where the data is available in included studies. We have clarified this in the analysis section of the methods as follows: “Where data is available, we will compare and contrast reported findings on preference and whether or not preference is aligned with improvement of outcomes of impact such as knowledge.”*

I was not fully sure why things like surveys measuring attitudes and opinions of people were not taken along in this review. (I am aware of the fact that guidance on the inclusion of such designs in reviews is scarce though-consider it a free offer to work towards a more comprehensive review type). Particularly, because in the RCTs authors tend to rely on self-reported measures, with LIKERT scales. Here, a comparison could sit with time, rather than comparator. Does people's attitude change when exposed to different formats over time?

*Author’s response: Thank you for this comment. The aim of this mixed-methods systematic review was to evaluate the effectiveness and acceptability of different evidence synthesis summary formats for CGDG members. For effectiveness we chose to restrict to RCTs as the most appropriate design to evaluate the effectiveness of the interventions in question. Therefore, observational studies like surveys were not included for the effectiveness question as they would not be appropriate to answer the question posed, as described in the methods section. We did not explicitly exclude RCT designs that had interventions with ‘repeated exposures,’ multiple timepoints for outcome assessment or longitudinal follow ups so if identified such studies will be included. However, these design seem unlikely in this context. While policy makers, clinicians, and methodologists could potentially be involved in multiple guideline development groups, this would likely be over quite an extended period of time and would likely have objectives related to the decision-making process and attitude change over time. RCTs exploring this seem unlikely and repeated surveys would not provide effectiveness data.*

*To explore opinions and attitudes, we included qualitative studies and mixed-methods studies with primary qualitative data collection. We choose to focus on primary qualitative data as this data in this context. In keeping with Cochrane guidance, we do not consider a questionnaire survey to be a qualitative study (* *https://training.cochrane.org/handbook/current/chapter-21* *). We prioritized inclusion of qualitative data from primary studies over free text from questionnaire surveys as we hypothesized primary data would be richer and thicker and thus more informative. We have clarified this in the methods section as follows: “We prioritized the inclusion of qualitative data from primary studies over free text from questionnaire surveys as we hypothesized primary data would be richer and thicker and thus more informative.”*

 Sometimes the arguments are not fully clear. In the introduction, attention is paid to problems complicating the conduct of systematic reviews. This is not the core focus of the project. The project is linked to the translation phase of turning review findings into evidence summaries. This is a matter of content, form and channels rather than the SR pre-track issues. It would therefore be more appropriate to highlight the diversity of actors involved in producing guidelines in relation to how communication and dissemination channels have changed because of that. I am a big fan of the authors discussing things like podcasts and multimedia accounts as examples. Naturally, this is linked to a society moving into multimodality and the limitations of numbers and narratives in reaching out to or working with different publics.

*Author’s response: Thank you for this feedback. We have edited the introduction to try to truncate the paragraph on systematic reviews and to try to relate more to the core focus of the project. The process of conducting a systematic review is related to the time and resources the team will have to create the summary formats themselves so we have largely kept the information about the ‘pre-track’ problems. Furthermore, we are focusing on multiple types of evidence syntheses and the landscape has recently evolved in this regard. This also directly relates to the search strategy used and inclusion criteria.*

 The PICO would perhaps work better if clinicians is changed into professionals. It would make it relevant to a broader target group.  Also, the comparator could also be no summary at all, for example, reading findings from reviews straight into the guideline procedure. Why are these explicitly excluded from the review?

*Author’s response: Thank you for this question. We have changed clinicians to healthcare professionals to be more inclusive. We have excluded no summary at all as a comparator because we do not think it is appropriate to compare the reading experience of a summary format versus none. We have added text to the ‘Quantitative systematic review’ section: Studies where the comparison is no intervention (e.g., the full-text of an evidence synthesis manuscript) will be excluded. Producing a full text of an evidence synthesis without some form of summary is not ‘usual care’. Internationally, organisations, journals and reporting guidelines would consider a summary to be a mandatory component of any report/peer review manuscript produced (e.g., Cochrane, PRISMA guidance, etc.).  Therefore no form of summary or abstract ‘is not an appropriate comparator*

 Do the authors only include qualitative studies where an intervention is evaluated by participants in terms of meaningfulness or applicability? Or do they also include qualitative studies where attitudes, viewpoints and options for form/channel/content are discussed from a more theoretical point of view, or in pilot cases? It is not clear where the selection sits on the qualitative side, but the data collection part suggests the first option.

*Author’s response:  Thank you for this question. We have chosen the SPiDER approach for the qualitative evidence synthesis as we are not restricting qualitative studies to those that evaluate an intervention in terms of meaningfulness of applicability. Our evaluation outcomes include the ‘Views, attitudes, opinions, experiences, perceptions, beliefs, feelings, understanding’ of participants. The information in the data collection section regarding the qualitative data is just to provide a summary overview of the study’s main findings (outcomes) to help with descriptive analyses. We edited the text to emphasize that NVivo12 coding will be conducted ‘in parallel’ (rather than ‘however, included’ as previously worded).*

 Perhaps the authors may want to bring a rationale for why they want to limit the formats to textual and audio-visual productions, as there are many more forms available through which evidence is and can be communicated (such as installations, theatre, etc.) Perhaps these forms haven't been studied extensively in the context of guidelines, but in a protocol phase we are not sure what to find yet :-).

*Author’s response: Thank you for this suggestion. We did not list any exclusion of formats explicitly, nor did we limit the formats to textual or audio-visual productions. In the list provided, we used e.g. ‘e.g., visual, audio, text based’ (for example) to list potential summary modes we may find rather than ‘i.e.’ (that is) to explicitly list summary modes we are restricting our search to. Our search strategy included specific formats that we thought we might find but we would not exclude other types of communications that weren’t explicitly listed. We have added text to the ‘quantitative systematic review’ section to clarify this ‘We will not exclude a summary format if it is one that we did not explicitly list in our search strategy (Table 3).’*

 I would find it more practical if the authors would discuss their position towards critical appraisal and how to structure this instead of putting it in the data collection part as an extraction category (it is not clear what will actually be extracted for what purpose). Also, critical appraisal checklist for primary studies have a different purpose than GRADE and CONQUAL that work on a review level (which is clearly not the focus here when talking about the pool of studies included). I think the authors need to spell the two out in more detail, as they are used in different phases of the review process.

*Author’s response: Thank you for this suggestion. In reporting this protocol, we have followed the JBI manual and as such the ‘bias and quality assessments’ subheading should be it’s own unique section and does not fall under the data collection section. The JBI critical appraisal tools are mentioned in the data collection section as they are a part of the standard data-extraction form that will be used by the reviewers. These should be two separate sections; apologies if there was a formatting error. We have edited the text within the ‘data collection’ and ‘bias and quality assessments’ sections to try to clarify what will be extracted for what purpose and to clarify the GRADE/ConQual concern. The text changed in the data collection now reads: ‘The following data information will be extracted using the pilot-tested standardized data-extraction form’ and ‘Data from the domains listed within the JBI critical appraisal tools for qualitative and quantitative studies.’*

*We agree that the critical appraisal checklist for primary studies have a different purpose than GRADE and CONQUAL. We have reworded the information in the ‘bias and quality assessments’ section to clarify that the JBI checklists are for individual studies AND there is no checklist recommended for the final assessment in the entirety of evidence (similar to the GRADE and CONQUAL purposes) for a mixed methods systematic review.*

 Would quality of a study not rather fit a sensitivity analysis rather than a subgroup analysis? What purpose would it have as a subgroup in the review? It has disappeared in the mixed method part as a subgroup analysis, so wouldn't mention it as a subgroup in other parts of the text.

*Author’s response: Thank you for this question. We recognize that the text in the quantitative analysis section was unclear. We have edited the text, deleting the for example list and restricting it only to the subgroups of interest (participant group or summary format type). We note subgroup analyses for participant groups or intervention types as these involve comparisons across the different groups. There are no planned sensitivity analyses for study quality as there will likely be far too much heterogeneity in outcomes (and the studies in general) to be able to compare the different methods.*

 What do the authors mean with the phrase "If textual pooling is not available, a narrative summary will be presented." Is a narrative summary not already a textual pooling? Unclear.

*Author’s response: Thank you for this question. Apologies for this issue. We have edited the text accordingly.* ‘ *If we are unable to pool findings together (i.e., create and present categories), likely due to an insufficient number of studies identified, a narrative summary will be presented.’*

 While configuration might have different meanings, it is generally not commonly used as a metaphor for a separate type of synthesis. Configuration would make the different parts unrecognizable in the whole. I don't think meta-aggregation would achieve this level of integration. Perhaps integration is better than configuration here.

*Author’s response: Thank you for this suggestion. We have reworded the mixed methods synthesis section to make our approach more clear and have added a references to the specific sections of the JBI manual that we are referencing regarding configuration and the thematic analysis (chapter 8 and section 8.2 for methodology; not section 2 which is for SR of qualitative evidence which details the meta-aggregation approach). We are following JBI’s guidance for MMSRs, and have chosen to use a convergent segregated approach. We have added additional text to clarify that ‘There are several methods for integrating qualitative and quantitative evidence syntheses26, [2] in a convergent segregated MMSR. We will use a thematic synthesis method for integration which groups together similar codes, develops descriptive categories (or themes) to create an overall summary of findings. ^41, 42’ ^Meta-aggregation is solely used for the qualitative analysis, not for the mixed methods synthesis which includes the integration.*

 Also, thematic synthesis is not the same as meta-aggregation, and line by line coding is certainly not a part of meta-aggregation. I believe the authors need to make an informed choice between qualitative evidence synthesis approaches and then follow the guidance through.

*Author’s response: Thank you for this comment. Apologies for the confusion but we are not using a meta-aggregation approach for the mixed methods synthesis – only for the qualitative analysis which is done prior to the integration of the two separate streams of evidence. We have edited the mixed methods synthesis section to further clarify our methodological approach which is in accordance with chapter 8 of the JBI manual.*
